# Correspondence

**Published:** 1860-08

**Authors:** C. N. Woodward


### Correspondence.

Messrs. Editors :—It is asserted by good authority, that by the mouth of two or three witnesses every word shall be established. Now I do not expect to add one ray of light or information, after such as have spoken on the subject of which I am about to say a few words, by way of adding my testimony. In February, 1860, at the meeting of the Mississippi Valley Association, I heard Dr. W. H. Atkinson, of Cleveland, Ohio, on the use of the mallet for filling teeth, and I endeavored to listen with that consideration due to a man of his experience, ability and standing. But I must here frankly acknowledge that I looked on the subject with a great degree of distrust; and although I frequently ruminated the subject in my mind, I had not the courage to adopt it for fear of periostitus—horror to the patient of being struck in the mouth with a formidable hammer, sledge, or mallet.

I concluded to wait for the facts, or at least until I could have ocular demonstration at the hand of some experienced and expert operator. Fortune favored me, for being in Cincinnati in the month of May following, I called on Prof. Taft, in whom I have implicit confidence as an operator. The subject of the mallet was broached by him, and I took the liberty to make some inquiry, which by the way, I always feel at liberty to do when in the company of one so well qualified to instruct, and so urbane a gentleman. And to have the subject matter demonstrated, I had Prof. Taft remove a filling of large capacity from an inferior molar, that was placed there by Dr. C. C. Allen, of New York, some twenty-five years ago; and at that time, under the then existing administration, was considered a first class filling. It certainly has done good service, but the tooth had commenced to decay at or near the filling, and the dentine was quite sensitive to the touch of the excavator. The Prof. removed the filling, and excavated in his thorough manner; all being ready, then came the mallet, wielded not by the heavy hand of a blacksmith, but by the silken hand of a fair lady. And the said tender tooth was filled solid, perfect and complete, with the greatest facility, and to my astonishment, with great ease to myself, without any real pain; nor has there been ever since any soreness or inconvenience from the tooth or surrounding parts.

I then and there in my own mind resolved on the use of the mallet, and have used it in all practicable cases since (and nearly all cases with suitable instruments can be filled with the mallet) to my entire satisfaction, and I believe to the satisfaction of my patients, without a single exception or bad result, so far as I know; with greater facility and ease to myself and to my patients. As I have already spun my yarn too long, and it not being necessary for me to enter into a description of the modus operandi, it having been so well and fully explained by one better qualified for the task, I will close by saying to all who wish to excel, try it.

Respectfully,

C. N. Woodward.

P. S. I use the Crystal Gold Foil in almost every case.

